# In Vitro Assessment of Cadmium Bioavailability in Chinese Cabbage Grown on Different Soils and Its Toxic Effects on Human Health

**DOI:** 10.1155/2015/285351

**Published:** 2015-06-18

**Authors:** Rukhsanda Aziz, Muhammad Tariq Rafiq, Zhenli He, Di Liu, Kewang Sun, Yang Xiaoe

**Affiliations:** ^1^Ministry of Education Key Laboratory of Environmental Remediation and Ecological Health, College of Environmental and Resource Sciences, Zhejiang University, Hangzhou 310058, China; ^2^Department of Environmental Science, International Islamic University, Islamabad 44000, Pakistan; ^3^Indian River Research and Education Center, Institute of Food and Agricultural Sciences, University of Florida, Fort Pierce, FL 34945, USA; ^4^Department of Thyroid and Breast Surgery, Zhejiang Provincial People's Hospital, Hangzhou 310014, China

## Abstract

The minimum concentration of cadmium (Cd), by Chinese cabbage grown on Cd contaminated soils that can initiate toxicity in human liver cells using in vitro digestion coupled with Caco-2/HL-7702 cell models was studied. Cadmium bioaccessibility in the gastric phase for yellow soil (YS) cabbage (40.84%) and calcareous soil (CS) cabbage (21.54%) was significantly higher than small intestinal phase with the corresponding values of 21.2% and 11.11%, respectively. Cadmium bioavailability was higher in YS cabbage (5.27%–14.66%) than in CS cabbage (1.12%–9.64%). Cadmium concentrations (>0.74 *μ*g) transported from YS and CS cabbage were able to induce oxidative (MDA, H_2_O_2_) stress by inhibiting antioxidant (SOD, GPx) enzyme activities in human liver cells (HL-7702). Additionally the study revealed that the ingestion of Cd contaminated Chinese cabbage grown in acidic soil (yellow soil) weakened the antioxidant defense system under all levels of contamination (2, 6, and 9 mg·kg^−1^) which ultimately escalated the oxidative stress in liver cells; however, in case of CS cabbage, a marked oxidative stress was observed only at 9 mg kg^−1^ Cd level of soil. Therefore, it is necessary to monitor Cd concentrations in leafy vegetables grown on acidic soils to minimize human health risk.

## 1. Introduction

The accumulation of heavy metals and metalloids in agricultural soils is of increasing concern due to the food safety issues and potential health risks as well as its detrimental effects on soil ecosystems [1, 2]. Like other industrial countries heavy metal contamination of agricultural soils has become an increasingly serious environmental issue in China [3, 4]. Approximately 20% of farm lands in China are contaminated with heavy metals and Cd contamination accounts for more than 1.3 × 10^5^ ha of the total affected area [5]. The introduction of Cd from soils into the food chain is of concern because Cd is readily taken up and translocated into above ground portions of the plant [6]. In general, the phytoavailability of Cd depends on different physical and chemical characteristics of the soils and crop cultivars [3, 4, 7]. Furthermore, heavy metal accumulation in vegetables is directly proportional to the metal concentration in the contaminated soil. Therefore, crops cultivated from Cd contaminated soils may be unsuitable or even detrimental for animal and human consumption [8].

Ferruzzi and Blakeslee [9] reported that increased vegetable consumption is associated with a decreased cancer risk, emphasizing increased consumption of vegetables. However, vegetables especially leafy vegetables have the ability to accumulate higher concentrations of Cd than other crops [10]. Chinese cabbage also known as Pakchoi (*Brassica chinensis *L.) is a common and widely consumed vegetable in China, which has a rapid growth rate of about 2-3 months from sowing. Yan et al. [11] recently measured Cd concentrations in Chinese cabbage grown in a metal contaminated area and reported that Cd in Chinese cabbage has a high contribution to the total daily intake of Cd for human. Therefore, it is necessary to control Cd concentrations in Chinese cabbage, especially in the edible parts to ensure food safety. Consequently, people could be at risk of adverse health effects from consuming vegetables grown in Cd contaminated soils. Persistent intake of Cd even at a low concentration adversely affects humans and animal's health [12]. Hence, there is an urgent need to determine the bioaccessibility, bioavailability, and toxicity of Cd from Chinese cabbage grown on contaminated soils to assess the human health risk.

In vitro methods can be used to assess the bioaccessibility, bioavailability, and toxicity of Cd for human, for example, from soil, food, and so forth [13, 14]. The technique measures the fraction of a metal which is solubilized from a sample under simulated gastrointestinal conditions and which therefore is available for absorption (bioaccessibility) [15], and after absorption it has the potential to reach the circulatory system and exert its toxic effects on the target organs (bioavailability). These in vitro digestion methods are alternatives to in vivo methods due to their low cost, energy saving properties, and time and their independence from physiological factors [15, 16]. Human intestinal epithelial cell line (Caco-2) has been used in the development of in vitro research and mimics the process of intestinal cell retention and transport. In this study, a combination of in vitro digestion/Caco-2 and HL-7702 cells was used in to assess the bioaccessibility, bioavailability, and toxicity of Cd from contaminated Chinese cabbage.

After absorption Cd is rapidly transported to its target organs (liver and kidney), causing several metabolic and histological alterations. Recent reports suggest that ROS generation associated with Cd exposure leads to oxidative stress, which is responsible for Cd induced apoptosis and toxicity in normal human liver cells (HL-7702) [17]. Chronic exposure to Cd increases an imbalance among oxidants and antioxidants of the cell tissues revealing oxidative damage in target organs [17, 18]. However, minimal attention has been paid to study Cd induced oxidative stress and ultrastructural changes in liver cells using bioaccessible fractions from in vitro digested vegetables grown on contaminated soils.

Predicting the bioavailability and mobility of trace metals in soils has been an important issue in agricultural and environmental studies. Investigations have been conducted on heavy metal uptake from soil to edible parts of plants [3, 4, 19, 20] but a few studies have reported the bioaccessibility and bioavailability of heavy metals from vegetables to human [1, 14, 21]. Moreover, information is lacking on transfer of Cd from edible parts of crops to human and its toxicity in the target organs. To the best of our knowledge, this is the first study reporting the transfer of Cd from two different soil types to Chinese cabbage and its subsequent effects on bioaccessibility, bioavailability, and toxicity in human.

The objectives of this study were to (i) assess the impact of different textured soils on Cd accumulation in Chinese cabbage; (ii) estimate the Cd concentrations in edible shoots of Chinese cabbage and its bioavailability in Caco-2 cell model using in vitro digestion; and (iii) confirm the minimum concentration of Cd that can initiate toxicity in human liver cells using in vitro digestion coupled with Caco-2/HL-7702 cell models.

## 2. Materials and Methods

### 2.1. Chemicals and Reagents

Cd (NO_3_)_2_ was purchased from Sinopharm Chemical Reagent Co., Ltd. (Shanghai, China). Porcine pepsin, pancreatin, bile salts, and 3[4, 5-dimethylthiazol-2-yl]-2, 5-diphenyltetrazolium bromide (MTT) were purchased from Sigma-Aldrich (St Louis, MO). Dulbecco's modified Eagle medium (DMEM with glucose 4.5 gL^−1^), trypsin-EDTA, phosphate-buffered saline (PBS), and fetal bovine serum (FBS) were purchased from Gibco Life Technologies (Grand Island, NY). All enzymes testing kits were purchased from Nanjing KeyGen Biotech. Co., Ltd. (Nanjing, China).

### 2.2. Ethics Statement

The soils used in the current study were agricultural soils. No specific permissions were required for the described locations. We confirm that the field studies did not involve endangered or protected species. The Caco-2 and HL-7702 cell lines were obtained from the Institute of Biochemistry and Cell Biology (SIBS, CAS Shanghai, China) and used in assays at passages 22–38.

### 2.3. Soil Collection and Analysis

In this study, we selected two different textured Chinese soils. Samples of yellow (Periudic Argosols) soil and calcareous (Calcaric Regosols) soil were collected from depth of up to 20 cm from the top horizon in Huzhou (119°14′–120°29′E, 30°22′–31°11′N) and Ya'an county (102°37′–103°12′E, 29°23′–30°37′N), respectively. The soils were air-dried and ground to pass through a 2 mm sieve before laboratory analysis. Selected physicochemical properties of these soils are presented in (Table 1).

Yellow and calcareous soil samples were spiked with Cd as Cd(NO_3_)_2_ dissolved in an aqueous solution at the loading rates of 2, 6, and 9 mg Cd kg^−1^ soil along with background value (CK). All the Cd-spiked soil samples were aged for about two years prior to usage for containerized experiments and Cd analysis.

### 2.4. Greenhouse Experiment

A greenhouse experiment was conducted by growing Pak choi (*Brassica chinensis* L.) in pots during March-April 2013 at Zhejiang University, Hangzhou, China. Seeds were germinated in dark at 25°C and transplanted into quartz sand bed to establish seedlings. After 20 days of sowing, morphologically uniform seedlings were selected and transplanted into plastic pots diameter of 18 cm and height of 17 cm filled with 3 kg of soil. The experiment was conducted with three replicates arranged in a greenhouse under controlled conditions of 16 h of light at 30°C and 8 h of dark at 22°C, and the plants were monitored daily and watered as necessary.

After 32 days, Chinese cabbage plants were harvested from each pot and separated into root and shoots (including stems and leaves). Shoots were first washed with tap water and then with ultrapure distilled water, to remove all visible soil particles. Filter paper was used to absorb any remaining water. Plant shoots (1.5 g) were dipped in 4.5 mL of ultrapure water and heated for 30 min in 90°C water bath, based on the Chinese dietary reference intakes [22]. The cooked plant samples were homogenized in a polytron homogenizer, and the homogenate was frozen and lyophilized before analysis via the in vitro digestion/Caco-2/HL-7702 cell model.

### 2.5. Total Cd of Soil and Plant

Soil sample (0.2 g) was digested with HNO_3_–HClO_4_–HF (5 : 1 : 1) [19]. Similarly, 0.25 g of cooked lyophilized plant homogenate was digested with HNO_3_–H_2_O_2_ (4 : 1). After cooling, the digest was transferred to a volumetric flask and diluted with distilled water up to 30 mL. Concentration of Cd in the filtrate was determined using inductively coupled plasma-mass spectrometry (ICP-MS, Agilent 7500a, Agilent Technologies, CA, USA) following standard procedure. The ICP-MS was operated at the following conditions: the carrier gas flow 0.95 L min^−1^, the auxiliary gas flow 0.89 L min^−1^, the plasma gas flow 15 L min^−1^, and the radio frequency power at the torch 1.2 kW [4].

### 2.6. Cadmium Bioavailability Assays

#### 2.6.1. In Vitro Digestion

The in vitro digestion method simulating the human gastrointestinal tract included gastric and small intestinal phases [23, 24] with a few modifications. Briefly, 15 mL of saline buffer (140 mM NaCl, 5 mM KCl) was added in 5 g of cooked lyophilized plant sample and pH was adjusted to 2 with 6 M HCl. For gastric digestion the sample was mixed with 0.5 mL of pepsin solution (0.2 g pepsin in 5 mL of 0.1 M HCl) and incubated on a shaking water bath at 37°C for 2 h. After 2 h of incubation the pH of digest was adjusted to 5.0. Aliquots (3 mL) were removed by syringe and filter (0.45 *μ*m); then the gastric digestion samples were stored at 4°C, prior to the total Cd concentrations of the sample by ICP-MS. To mimic small intestinal digestion, 2.5 mL of pancreatin-bile solution (0.45 g of bile salts and 0.075 g of pancreatin in 37.5 mL of 0.1 M NaHCO_3_) was added and the digester was then incubated in a shaking water bath at 37°C for 2 h. To stop the intestinal digestion and to separate the bioaccessible fraction, the digester was cooled in ice for 10 min, and then the pH was adjusted to 7.2 with 0.5 M NaOH. For Cd uptake assays with Caco-2 cells, the bioaccessible fraction obtained was heated at 100°C for 4 min to inhibit the proteases. The gastrointestinal digest was centrifuged at 3500 g for 1 h at 4°C. Prior to addition of plant bioaccessible fraction to the cells, glucose (5 mM) and HEPES (50 mM) were added in order to make it similar to culture media. The supernatant (bioaccessible fraction) was analyzed for Cd content in small intestinal phase and used in cell uptake assays.

#### 2.6.2. Cell Culture

Caco-2 and HL-7702 cell lines were normally cultured in 25 cm^2^ flasks (Corning Inc., NY, USA) and maintained in high glucose (4.5 g/L) DMEM, supplemented with 20% (v/v) fetal bovine serum, 1% (v/v) nonessential amino acids, 4 mM L-glutamine, and 1% (v/v) antibiotics solution (complete medium). The cells were incubated in a cell culture incubator (Heraeus, BB15, Germany) at 37°C, 5% CO_2_ in a humidified atmosphere and the medium was changed every 48 h. Monolayers were subcultured by treatment with 0.25% trypsin-EDTA solution. For Caco-2 cells, 5 × 10^4^ Caco-2 cells cm^−2^ in 1.5 mL of complete DMEM and 2.5 mL of complete DMEM were added in the basal compartment. For Cd toxicity assays HL-7702 cells were seeded in 6-well culture plates (Costar Crop, NY, USA) at a density of approximately 5 × 10^3^ cells/well. The medium was changed every 2 days, and the Caco-2 and HL-7702 cell cultures were maintained for 21 and 9 days, respectively, to reach the stationary growth phase and to allow for maximal functional differentiation. Subsequently, the integrity of Caco-2 cells monolayer was checked by measuring transepithelial electrical resistance (TEER) using a Millicell-ERS meter (Millipore Corporation, Bedford, MA, USA) according to the manufacturer's protocols. Only those filters that had TEER values >250 Ωcm^2^ at the beginning and the end of the experiment were included. The monolayer used in this study exhibited adequate TEER values 520–610 Ωcm^2^.

#### 2.6.3. Cd Uptake (Retention and Transport) by Caco-2 Cells

Before the Caco-2 cell uptake experiment, the DMEM was removed from each well and the cell layer was washed three times with phosphate-buffered saline (PBS) at a temperature of 37°C and pH7 to remove any unattached cells. Afterwards 2.5 mL of standard transport solution (130 mM NaCl, 10 mM KCl, 1 mM MgSO_4_, 5 mM glucose and 50 mM HEPES, pH 7.4) was added into the basolateral compartment while apical compartment was filled with 1.5 mL of Chinese cabbage small intestinal bioaccessible fraction.

After 2 h incubation, basolateral compartments were collected to determine the Cd transport across the monolayer as well as to check the toxicity in the HL-7702 cells via toxicity assays later in the study. The apical chamber cell monolayer was washed with phosphate-buffered solution (PBS) at pH 7 to remove nonspecifically attached minerals and residual medium. The cells were lysed by the addition of 1 mL deionized water in the well and then harvested. Caco-2 cell lysate was used for Cd retention analysis.

The concentrations of Cd in cell retention (Cd concentration in the Caco-2 cell monolayer), the transport solution (Cd collected from basolateral compartment), and the gastric and small intestinal digestion solution (Cd bioaccessible fraction) from cooked Chinese cabbage samples were determined using the ICP-MS. Bioaccessibility of Cd at gastric and small intestinal phases was analyzed according to Oomen et al. [25] as follows:

(1) Bioaccessibility %=Cd  mobilized  from  plant  sample  during  digestion  μgCd  present  in  plant  sample  before  digestionμg ×100.

### 2.7. Cadmium Toxicity Assays by HL-7702 Cells

To assess the Cd toxicity, HL-7702 cells cultured in 6-well plates were exposed to the transport solution collected from Cd uptake experiment, discussed earlier. The cell cultures were incubated for 3 h at 37°C, 5% CO_2_, and 95% relative humidity [26]. After incubation the cells were washed thrice with PBS to remove extracellular bound Cd. The HL-7702 cell monolayer was lysed by the addition of 1 mL of 0.25% trypsin-EDTA and then harvested. Cytological and biochemical assays were carried out on the HL-7702 cell lysate and culture medium for determination of markers of cell damage and TEM.

#### 2.7.1. Cytotoxicity Assay

*(1) MTT Assay*. The effect of Cd on cell viability was determined by the MTT assay. MTT assay provides a quantitative measure of surviving and/or proliferating cells by determining the amount of formazan crystals produced by metabolic activity in treated versus untreated control cells. Positive control groups consist of cells in media, which are processed identically and incubated simultaneously as treated groups. MTT viability assay was performed according to Aziz et al., 2014 [18]. After 3 h incubation to Cd containing transport solution, cell viability was expressed as a percentage of the positive control group. Cell MTT response (% of control) was calculated by the following equation:

(2) $$\begin{array}{c} \%\;\;\text{of}\;\text{Control}\;=\frac{{\text{Absorbance}}_{\text{Treatment}}}{{\text{Absorbance}}_{\text{Control}}}\times 100. \end{array}$$

*(2) LDH Release*. Cadmium cytotoxicity from HL-7702 cells was also monitored by LDH release into the culture medium after 3 h of exposure to Cd containing transport solution. The LDH release into the culture medium was determined with a commercially available kit (Jiancheng Biochemical Co., Ltd.) (Nanjing, China) according to the manufacturer's protocol. The change in the absorbance was recorded at 440 nm using a microplate spectrophotometer system (Bio-Rad-680, Bio-Rad, USA). LDH release (% of control) was calculated from the following equation:

(3) $$\begin{array}{c} \%\;\text{of}\;\text{Control}=\frac{{\left(U_{\text{LDH}}/L\right)}_{\text{Treatment}}}{{\left(U_{\text{LDH}}/L\right)}_{\text{Control}}}\times 100. \end{array}$$

#### 2.7.2. Lipid Peroxidation Assay

Lipid peroxidation (LPO) was evaluated by measuring malondialdehyde (MDA) production according to the thiobarbituric acid (TBA) method with a commercially available kit (Jiancheng Biochemical Co., Ltd., Nanjing, China). MDA concentration was calculated by the absorbance of TBA reactive substances at 532 nm. The result was expressed as nmol MDA per mg cell protein.

#### 2.7.3. Antioxidant and Oxidant Enzymes Assays

The changes in the cell antioxidant (SOD, GPx) and oxidant (H_2_O_2_) enzyme activities were measured using respective cellular activity assay kits (Jiancheng Biochemical Co., Ltd., Nanjing, China). Enzyme activities changes were performed according to the manufacturer's protocol. SOD and GPx activities were expressed in terms of international units per mg cell protein. H_2_O_2_ enzyme activities were expressed as mmol per mg cell protein, respectively.

#### 2.7.4. Transmission Electron Microscopy

To morphologically observe the alterations in Cd-treated HL-7702 cells, an ultrastructural analysis was performed following the procedure of Shen et al. [27]. Briefly HL-7702 cells were harvested from 6-well culture plates, fixed in 4% (v/v) glutaraldehyde, and postfixed in 1% (v/v) osmium tetroxide. Then the samples were dehydrated by being treated with a graded series of ethanol followed by acetone. Hereafter, the samples were rinsed and impregnated with Spurr's resin. The ultra-thin sections were prepared and mounted on copper grids for viewing in the JEM-1230 transmission electron microscope (TEM) at an accelerating voltage of 60.0 kV.

### 2.8. Quality Control of Cadmium Analysis

Quality assurance and quality control (QA/QC) for Cd in soil and Chinese cabbage shoots (soil GSBZ 50013-88 and plant GBW-07402), respectively, were included in the digestion approved by General Administration of Quality Supervision, Inspection and Quarantine of the People's Republic of China (AQSIQ) and National Center for Reference Materials. The analytical results showed that the recoveries for SRMs were nearly 93.7% and 98%, respectively.

### 2.9. Statistical Analysis

Data were expressed as means ± S.D. and analyzed using SPSS software version 18.0 (SPSS Inc., Chicago, USA). Analysis of variance (ANOVA) was applied, with Duncan's post hoc test, to compare the various means of each series of experiments from control. A *P* value of less than 0.05 was considered to be significant.

## 3. Results

### 3.1. Characteristics of Soils

Soils used in the current study varied widely in their physicochemical properties (Table 1), which can influence the overall accumulation of Cd in Chinese cabbage shoots. Soil pH range was strongly acidic (4.92 for YS) to mild alkaline (8.11 for Cs). Cation exchange capacity (CEC) ranged from 12.06 cmol kg^−1^ to 26 cmol kg^−1^ for YS and CS, respectively. Total organic matter was 11.6 g kg^−1^ for YS and 22.6 g kg^−1^ for CS. Total Cd and Zn concentrations (background value) in YS were 0.47 and 5.10 mg kg^−1^, while for CS 0.96 and 33.9 mg kg^−1^, respectively. Fe (II) in soil (background value) was ranged from 25.4 to 36.7 mg kg^−1^ in yellow and calcareous soil, respectively.

### 3.2. Accumulation of Cd in Chinese Cabbage

**Figure 1:**
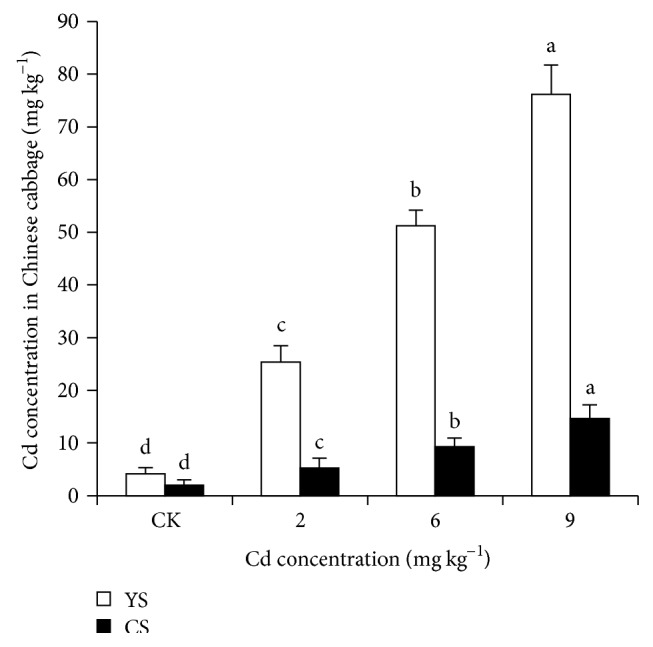
Cadmium concentration (mg kg^−1^ DW) in Chinese cabbage shoots grown under different Cd loading rates in yellow soil (YS) and calcareous soils (CS). The results are expressed as mean ± SD. Different letters indicate significant differences at *P* < 0.05 as calculated by Duncan's post hoc test, *n* = 3.

Cadmium concentration in the shoots of Chinese cabbage varied significantly among soils at different Cd levels and soil types (Figure 1). Cadmium concentration in Chinese cabbage shoots ranged from 4.13 to 76.16 mg kg^−1^ in YS and from 2.01 to 12.66 mg kg^−1^ in CS. Cd accumulation in Chinese cabbage shoots was affected by soil types, primarily due to the variation in Cd phytoavailability.

### 3.3. Bioaccessibility of Cd in Chinese Cabbage Shoots

**Figure 2:**
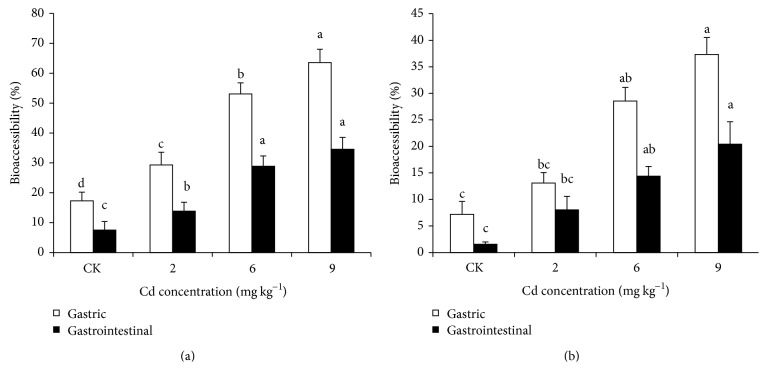
Effect of different Cd loading rates on the gastric and small intestinal bioaccessibility of Cd expressed as a percentage of the total concentrations in the Chinese cabbage shoots grown on YS (a) and CS (b). ^*^YS refer to yellow soil and CS calcareous soil. The results are expressed as mean ± SD with three replications. Different letters indicate significant differences at *P* < 0.05 as calculated by Duncan's post hoc test.

The amount of Cd solubilized after in vitro digestion is an indicator of bioavailability. Cadmium bioaccessibility in gastric and small intestinal phases significantly affected the increase in Cd loading rates (Figures 2(a) and 2(b)). Cadmium bioaccessibility in gastric phase (17.32–63.62%) and in small intestinal phase (7.50–34.54%) were found to be significantly higher in YS cabbage than CS cabbage with the corresponding values of 7.21–37.32% and 1.57–20.43%, respectively. Cadmium bioaccessibility in both soils cabbage was highest in gastric phase than in small intestinal phase.

### 3.4. Bioavailability of Cd from Chinese Cabbage

The soluble fraction obtained from small intestinal digestion phase was used to conduct the Cd retention, transport, and uptake experiments in Caco-2 cell. Cadmium concentration in intestinal bioaccessible fraction added to Caco-2 cells (soluble added), in cell monolayer (retention), basal medium (transport), and percentages of uptake are reported (Table 2). Increasing Cd loading rates had a significant impact on Cd retention, transport, and uptake results. In YS and CS cabbage Cd retention was significantly increased from 0.38 to 72.15 *μ*g and from 0.01 to 5.28 *μ*g, respectively. Cadmium transport had a significant increase by 0.11–43.52 *μ*g and 0.00–2.20 *μ*g for YS and CS cabbage, respectively. Percentages of Cd retention, transport, and uptake calculated as the amount initially added (soluble added) indicated that, in YS and CS cabbage, Cd retention (4.09–9.14% and 1.05–6.80%), transport (1.18–5.51% and 0.06–2.84%), and uptake (5.27–14.66% and 1.12–9.64%) varied largely due to contrasting properties of the two soils.

### 3.5. Cd Affected cell Viability (MTT) and Stability (LDH Release) in HL-7702 Cells

**Figure 3:**
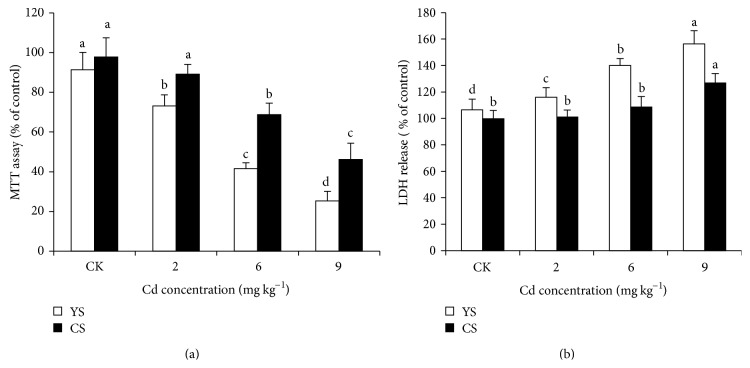
Effect of Cd transport solutions collected from human epithelial (Caco-2) cell monolayer on MTT viability assay (a) and LDH release (b) in HL-7702 cells after 3 h incubation. The results are expressed as mean ± SD. Different letters indicate significant differences at *P* < 0.05 as calculated by Duncan's post hoc test, *n* = 3.

The survival of HL-7702 cells with increasing Cd concentrations transported from the Caco-2 cells was measured by MTT cell viability and LDH release assays after 3 h incubation. In case of YS cabbage MTT assay (Figure 3(a)) revealed a significant difference in reduction of MTT between cell cultures exposed to increasing Cd concentrations and the control. However, in CS cabbage at higher Cd level 6 mg kg^−1^ (0.74 *μ*g transported Cd) and 9 mg kg^−1^ (2.20 *μ*g transported Cd) produced a significant decrease of 31% and 38%, respectively, with control.

In CS cabbage, LDH release to culture medium (Figure 3(b)) was nonsignificant in HL-7702 cells at lower Cd levels; however a significant increase of 27% in LDH leakage, compared to control cells, was detected in cells at 9 mg kg^−1^ Cd level (2.20 *μ*g transported Cd). A significant increase of 56% in LDH release occurred from YS cabbage (Figure 3(b)).

### 3.6. Cd Induced Lipid Peroxidation (MDA) in HL-7702 Cells

In HL-7702 cells YS and CS cabbage Cd induced an increase in MDA concentration by 57.44% and 28.23%, respectively (Table 3). MDA, a well-known indicator of lipid peroxidation, had a significant increase in CS cabbage grown at 6 mg kg^−1^ and above while YS cabbage had a significant increase at all Cd levels when compared with the control.

### 3.7. Effect of Cd on Antioxidant (SOD, GPx) and Oxidant (H_2_O_2_) Enzyme Activities in HL-702 Cells

Oxidant (H_2_O_2_) and antioxidant (SOD, GPx) enzyme activities in normal human liver cells (HL-7702) due to Cd transported from Caco-2 cells are reported (Table 3).

The results from oxidant enzyme activity indicated that hydrogen peroxide (H_2_O_2_) activity in YS and CS cabbage was increased by 67% and 52%, respectively, from its respective control. As can be observed in case of antioxidant enzymes, the SOD activities in the YS and CS cabbage had a percentage decrease of 43.5% and 20% while for GPx the decrease was about 38% and 21%, respectively, as compared to the control.

Generally, increasing Cd concentration affected the percentages of both enzyme activities in HL-7702 cells, but only in case of YS cabbage all enzymes had a significant change at all Cd levels while CS cabbage grown at 9 mg kg^−1^ significantly affected the oxidant and antioxidant enzyme activities. The abovementioned results indicated that above 0.74 *μ*g Cd concentrations transported from Caco-2 cells were able to initiate toxicity in normal human liver cells (HL-7702 cells).

### 3.8. Effects of Cd on Morphological Alterations in HL-7702 Cells

**Figure 4:**
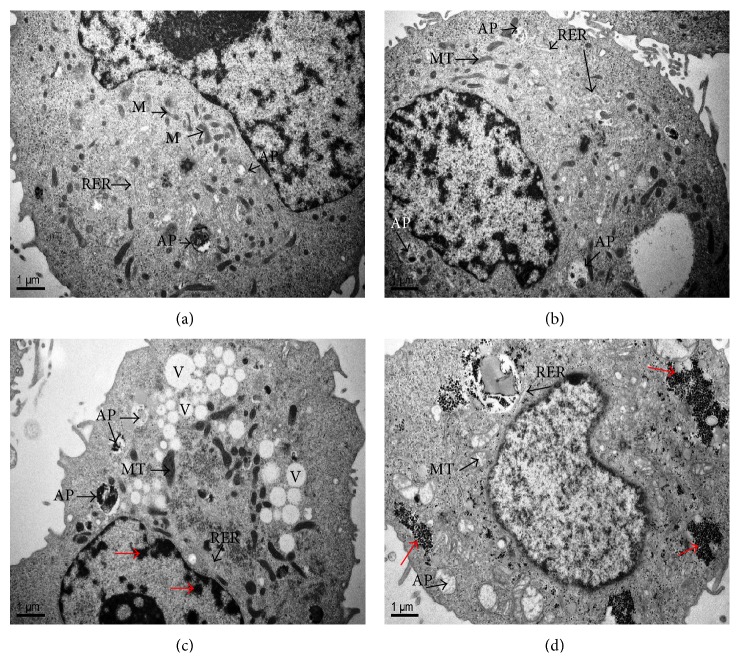
Electron microscopic graphs of HL-7702 cells exposed to YS and CS cabbage (CK and 9 mg kg^−1^ Cd) transport solutions collected from human epithelial (Caco-2) cells for 3 h: (a) HL-7702 cells of CS cabbage at the CK showing mitochondria (M), nuclei (N), and RER normal, (c) HL-7702 cells (CS cabbage) at Cd 9 mg kg^−1^ level showed more vacuolization (V), numerous swollen mitochondria, swollen RER with attached granules, condensation in heterochromatin (red arrows), and accumulation of protein inclusion bodies in autophagosomes (AP), (b) HL-7702 cells (YS cabbage) at the CK showing several mitochondria with invisible cristae, nuclei, and RER normal, autophagosomes with enclosed cytoplasmic contents, (d) HL-7702 cells (YS cabbage) at Cd 9 mg kg^−1^ level appeared with the remnants of several swollen and vacuolated mitochondria (M) conferring a spongy appearance, prominent RER with unbound granules, clusters of RER unbound granules (red arrows), and double membraned and lamellar autophagosomes (AP).

The TEM microscopic graphs of HL-7702 cells under a control and 9 mg kg^−1^ Cd stress from YS and CS cabbage are shown in Figures 4(a)–4(d). TEM micrograph of HL-7702 cells for CS and YS cabbage CK (Figures 4(a) and 4(b)), respectively, was characterized by round to ovoid nucleus with a small amount of heterochromatin. Nucleus is surrounded by cytoplasm having spherical to ovoid mitochondria (MT), rough endoplasmic reticulum (RER) with attached granules, and clear autophagosomes. CS cabbage TEM microscopic graphs of HL-7702 cells at 9 mg kg^−1^ Cd level (Figure 4(c)) illustrated the swelling in mitochondria (MT) with visible cristae, RER with bound granules, cytoplasmic vacuolization, and promotion in autophagosomes. However, the TEM for YS cabbage treated cells (at 9 mg kg^−1^ Cd level) (Figure 4(d)) appeared with the several swollen and vacuolated mitochondria (MT) conferring a spongy appearance, prominent RER with unbound granules, very clear clusters of RER unbound granules (red arrows), and double membraned and lamellar autophagosomes (AP).

## 4. Discussion

Due to recent rapid economic growth and industrial expansion, heavy metal contamination of agricultural soils in China has become a serious environmental concern [28, 29]. Cadmium (Cd) is generally considered a toxic metal where food is the major source of accumulation. Food chain contamination by soil cadmium (Cd) through vegetable consumption poses a threat to human health. An improved understanding of health risk posed to humans associated with Cd accumulation in Chinese cabbage is revealed.

### 4.1. Accumulation of Cd in Chinese

Different Cd loading rates significantly affected its accumulation in the shoots of Chinese cabbage (Figure 1). Phytoavailability of Cd is affected by physicochemical characteristics of soils [3, 4]. Liang et al. [30] reported that Cd concentration of spinach plants was highly dependent upon the soil pH being highest at pH 5.3, as changes in pH affect the sorption of Cd by soils and thereby its concentration in soil solution [31]. According to Kabata-Pendias, 2004, in well aerated (oxidizing) acid soils, several trace elements, especially Cd and Zn, are easily mobile and available to plants; however, in poorly aerated (reducing) neutral or alkaline soils, metals are substantially less available [32]. Moreover, it was observed that accumulation of the Cd in Chinese cabbage shoots grown in different soil types at 8 mg kg^−1^ ranged from 0.48 to 89.21 mg kg^−1^ [3, 4]. Phytoavailability of Cd in soil is also influenced by other factors including exposure time, soil texture, and organic matter contents in soil [3, 4, 31].

### 4.2. Cadmium Bioaccessibility from Chinese Cabbage Shoots

In the in vivo situation, Cd needs to be bioaccessible before it can be taken up by the enterocytes. We determined bioaccessible (gastric and small intestinal) Cd in the Chinese cabbage shoots by in vitro digestion. Our results revealed that Cd bioaccessibility of the cooked Chinese cabbage shoots from YS and CS was higher in gastric phase than in small intestinal phase (Figures 2(a) and 2(b)). These results are in agreement with the previous reports [14, 16, 21, 33]. The possible explanation was that most of the Cd accumulates in the vacuoles of plant cells, except that absorbed in the cell wall, so Cd is easily released from plant tissues [34]. Polysaccharides and proteins in the cell wall contain lots of oxhydryl, carboxyl, aldehyde, and phosphate groups, which have an affinity for metal ions. These groups could form coordinate bonds to lower the activity of the metal ions, inhibit ions across a cell membrane, decrease heavy metal content in the bioplast, and maintain a normal level of cell renewal [35]. So, in the gastric phase, most Cd was dissolved and a portion was still absorbed to plant tissues. That remaining portion was dissolved in the intestinal phase, due to the increase in pH and the addition of pancreatin and bile extracts. Higher bioaccessibility in small intestinal phase as compared to gastric phase could be explained by a higher degree of degradation of Cd binding to the food components in the intestinal juice as the pH for the pancreatic enzymes was optimal. The metal bioaccessibility of food is controlled by many factors, such as microfibers of crystalline cellulase and phytates, phytochelatins, nutritional characteristics, and microbial processes [36, 37].

### 4.3. Bioavailibility of Cd

In vitro digestion in combination with exposure of Caco-2 cell monolayers to supernatants of the YS and CS cabbage digests appears to be a promising model for studying the bioavailability of different Cd levels in Chinese cabbage. The Caco-2 model has been previously used to evaluate bioavailability of Cd in the raw and cooked leafy vegetables [21] and infant food [38] concluding that Caco-2 cell assays offer an improved indicator of bioavailability than solubility. The current study reported that monolayers incubated with bioaccessible fractions of YS cabbage had significantly higher Cd bioavailability (cellular uptake %) as compared to CS cabbage (Table 2). Previous studies reported that 25%, 4–15.9%, and 3.8–6.3% of Cd were up taken by Caco-2 cells from CdCl_2_ solution, leafy vegetables, and infant food [21, 38–40]. In our study, the Cd bioavailability ranged from 1.12% to 14.66% for the YS and CS cabbage; these values were both lower and higher than those reported in previous studies [21, 38, 39] but in YS cabbage and at higher Cd level (9 mg kg^−1^) in CS cabbage, this is not in agreement with the previously reported approximate 5% gastrointestinal absorption of Cd from food [41]. Differences in bioavailability of Cd from both YS and CS cabbage were maybe due to the soil type and Cd concentration from Chinese cabbage in the bioaccessible fraction. Cd bound to food components is indeed less water soluble than CdCl_2_, but enzymes and acids of the digestive tract may degrade these complexes making Cd available for absorption. Another possible explanation for the concentration-dependent Cd bioavailability in our study may be its chemical and physical properties related to essential metals such as iron (Fe) or zinc (Zn), which make Cd transported and taken up into the cells by ionic and molecular mimicry [42]. Whenever there is a deficiency of these essential elements, Cd absorption and toxicity are enhanced. However, other aspects need to be considered, for instance, effects of the bacterial flora in the human colon that potentially degrade a significant proportion of the dietary fibers. Cd bound to the fiber in vitro may thus be released during bacterial degradation and be absorbed in vivo.

The bioaccessibility of Cd does not always correlate with its bioavailability. The fractional small intestinal bioaccessibility of Cd after the Chinese cabbage digestion ranged from 1.6 to 34.5%, but the fractional bioavailability was generally much lower, varying from 1.12 to 14.70%. Cadmium in the YS cabbage had the highest bioaccessibility and bioavailability, which may indicate a higher risk of exposure due to both a higher Cd dose accessible to the intestinal cells and a higher cellular uptake which have the potential to reach the systemic circulation and cause toxicity to the target organs.

### 4.4. Toxicity Assays

The main objective of the present study was to assess the toxicity of bioavailable Cd from YS and CS cabbage to normal human liver cells (HL-7702 cells). Oxidative stress has been reported as an essential mechanism by which Cd induces toxicity. This Cd toxicity can be detected and measured within the HL-7702 cells in a variety of ways including the measurement of cytotoxicity assays (MTT, LDH), the concentration of MDA as an indicator of lipid peroxidation, oxidant enzyme like H_2_O_2_, and the activities of antioxidant (SOD and GPx) enzymes and finally to confirm all abovementioned toxicity assays transmission electron microscopy was conducted. To the best of our knowledge, this is the first study reporting the toxicity of bioavailable Cd, from Chinese cabbage grown on different textured soils, to normal human liver cells (HL-7702).

#### 4.4.1. Cytotoxicity Assays (MTT Viability and LDH Release)

Cytotoxicity induced by Cd exposure showed a dose dependent change in MTT and LDH release assays. The cytotoxicity assays employed revealed different profiles, as compared to LDH release, the MTT assay in YS cabbage being the most sensitive cytotoxicity assay showing statistically significant difference between the treated cells and the controls while CS cabbage had a significant change at 6 mg kg^−1^ Cd and above. These findings are in agreement with previous studies [18, 26]. The possible reason of this differential sensitivity can be explained by the different nature of each assay. The LDH leakage assay is based on the release of the enzyme into the culture medium after cell membrane damage whereas the MTT assay is mainly based on the enzymatic conversion of MTT in mitochondria [26]. It is thought that inhibition of the mitochondrial respiration induces active oxygen related cell death. Several investigations have demonstrated that the toxicity by Cd is associated with reactive oxygen species (ROS) [43] generated within the mitochondria and can also damage mitochondrial components [44]; therefore a cytotoxicity assay based on mitochondrial respiratory activity would present early signs of toxicity following exposure to a mitochondrial toxicant. Previous studies also reported that Cd may influence mitochondrial function [44]; therefore, mitochondrial injury may be a candidate mechanism of cell death.

#### 4.4.2. Effect of Cd on Antioxidant (SOD, GPx) and Oxidant (H_2_O_2_) Enzyme Activities in HL-702 Cells

Cadmium (Cd) is responsible for the generation of ROS and alteration of antioxidant enzymes activities in living cells [45]. Several studies demonstrate a relationship between Cd exposure and lipid peroxidation [46–48] indicating that Cd increased the hepatic level of malondialdehyde (MDA), a well-known indicator of lipid peroxidation and oxidative stress. A significant increase in MDA and H_2_O_2_ activity occurred due to Cd stress in HL-7702 cells (Table 3) which is in agreement with the findings of Jurczuk et al. [49] and Pillai and Gupta [50]. The Cd induced oxidative toxicity is multidirectional since it directly interrupts the activity of enzymes, a decrease in the level of glutathione, and the total pool of sulphydryl groups and depletion in the antioxidant enzyme activities [51]. Cd, through binding to the inner membrane, enhances lipid peroxidation and disturbs the integrity of mitochondrial membranes.

Cadmium is a nonredox metal that can indirectly cause oxidative stress by depleting cellular antioxidant enzyme system. To minimize Cd induced oxidative damage, organisms have developed antioxidative mechanisms triggered by an increased ROS production. SOD and GPx are important antioxidant enzymes that protect from oxidative stress via depletion of ROS. Physiologically, SOD converts O_2_^−^ to O_2_ and finally to H_2_O_2_ and thus protects against superoxide-induced damage [52] while GPx, in particular, is highly dependent on glutathione concentration. A significant decrease in the antioxidant (SOD, GPx) enzyme activities occurred in HL-7702 cells exposed to the YS cabbage's transport solution while from CS cabbage Cd stimulate SOD activity at lower level (<2 mg kg^−1^) but this stimulation was only transient and the SOD activity under high levels (>2 mg kg^−1^) of Cd decreased compared with that of the control; however, a significant reduction in GPx activity was observed at 9 mg kg^−1^ (Table 3). This differential change in the antioxidant enzyme activities from YS and CS cabbage may be concentration dependent.

### 4.5. Morphological Alterations in HL-7702 Cells

Indeed, low doses (<0.74 *μ*g) of Cd transported from the bioaccessible Chinese cabbage grown on different soils did not induce apoptosis and toxicity in HL-7702 cells, as revealed by cytotoxicity assays and enzyme assays. A deeper understanding of Cd hepatotoxicity requires the identification of its intracellular localization and targets. So we did the TEM of HL-7702 cells to observe the ultrastructural changes and to confirm the findings of all abovementioned toxicity assays. TEM had a certain degree of mitochondrial, RER, and nuclear and autophagic changes in HL-7702 cells when treated with YS cabbage (9 mg kg^−1^ Cd level) while for CS cabbage treated cells also showed little swelling in mitochondria, RER, and promotion in autophagy. All the ultrastructural alterations in treated cell cause the cell to stop functioning and lead to cell lysis. Our findings agree with the previous reports [53–55]. Consistently, we also provide evidence that the main intracellular target for Cd in vitro was autophagy, an adaptive and catabolic mechanism that involves the lysosomal compartment. The autophagic vesicles were barely detectable in untreated cells (Figures 4(a) and 4(b)) and rapidly increased upon Cd exposure (Figures 4(c) and 4(d)). Autophagy is a housekeeping process that ensures the turnover of damaged protein aggregates and organelles to maintain cell homeostasis [56]. The possible explanation for the upregulation of autophagy might be the binding of Cd to the sulfhydryl groups of proteins as well as the oxidative damage generated by this Cd in the cells [43, 57].

## 5. Conclusion

In conclusion, physicochemical properties of soil greatly influenced the bioavailability of Cd to Chinese cabbage and ultimately to Caco-2 cells. We may be able to avoid the dietary toxicity of Cd by selection of a proper soil type for Chinese cabbage cultivation. An important finding from the toxicity assays is that Cd concentrations (>0.74 *μ*g) transported from the bioaccessible fraction of Chinese cabbage are able to induce oxidative (MDA, H_2_O_2_) stress by inhibiting antioxidant (SOD, GPx) enzyme activities in human liver cells (HL-7702). The results indicated that the ingestion of Chinese cabbage grown on Cd contaminated acidic soil (YS) weakened the antioxidant defense system which ultimately escalated the oxidative stress in liver; however in CS cabbage the significant change was only observed at 9 mg kg^−1^ Cd level. This investigation also raises the possibility of simple soil remediation methods like liming and phytoextraction by using the coculture of Cd hyperaccumulator plants and leafy vegetables especially in acidic soils, to prevent further risk of Cd disease. Our study was an effort to estimate the Cd bioavailability and toxicity in human (Caco-2, HL-7702 cells) from Chinese cabbage grown on Cd contaminated soils. Further studies are required to investigate the biochemistry and mechanisms involved in the bioavailability and toxicity of Cd from Chinese cabbage to resolve remaining questions and establish proper therapeutics measures for chronic and acute intoxication of dietary Cd intake.

## Figures and Tables

**Table 1:** Physicochemical properties of soils.

Soil types	Yellow soil	Calcareous soil
OM (g kg^−1^)	11.6 ± 0.72	21.8 ± 0.09
CEC (cmol kg^−1^)	12.05 ± 0.61	26.00 ± 1.34
pH	4.92 ± 0.06	8.11 ± 0.43
Total N (g kg^−1^)	1.14 ± 0.07	1.46 ± 0.05
Total P (g kg^−1^)	1.72 ± 0.08	1.00 ± 0.03
Total K (g kg^−1^)	7.27 ± 0.19	9.53 ± 1.05
Total Cd (mg kg^−1^)	0.47 ± 0.02	0.96 ± 0.03
Total Zn (mg kg^−1^)	12.92 ± 0.85	33.9 ± 0.48
Fe(II) (mg kg^−1^)	25.4 ± 1.33	36.7 ± 2.18

**Table 2:** Bioavailability: total Cd retention, transport, and uptake by Caco-2 cells from cooked Chinese cabbage shoots.

Soils	Cd levels	Bioaccessible fraction^¤^ (*μ*g)	Retention (*μ*g)	Transport (*μ*g)	Retention%	Transport%	Cellular uptake%
YS^*^	Ck	9.29 ± 2.85^c^	0.38 ± 0.07^d^	0.11 ± 0.06^c^	4.09 ± 0.14^c^	1.18 ± 0.21^c^	5.27 ± 0.74^c^
	2	105.40 ± 6.04^c^	8.12 ± 0.99^c^	4.01 ± 0.50^c^	7.70 ± 0.40^b^	3.80 ± 0.48^b^	11.51 ± 0.59^b^
	6	444.20 ± 51.95^b^	39.08 ± 3.60^b^	21.31 ± 2.16^b^	8.80 ± 0.94^ab^	4.80 ± 0.45^a^	13.60 ± 0.81^a^
	9	789.20 ± 191.86^a^	72.15 ± 2.97^a^	43.52 ± 5.96^a^	9.14 ± 0.94^a^	5.51 ± 0.50^a^	14.66 ± 0.90^a^

CS^*^	Ck	0.95 ± 0.24^d^	0.01 ± 0.00^c^	0.00 ± 0.00^c^	1.05 ± 0.02^c^	0.06 ± 0.02^c^	1.12 ± 0.17^c^
	2	12.57 ± 1.02^c^	0.55 ± 0.05^c^	0.19 ± 0.04^c^	4.38 ± 0.65^b^	1.15 ± 0.03^b^	5.89 ± 0.65^b^
	6	40.24 ± 2.76^b^	2.01 ± 0.09^b^	0.74 ± 0.21^b^	4.99 ± 0.31^b^	1.84 ± 0.0.43^a^	6.83 ± 1.08^b^
	9	77.60 ± 8.11^a^	5.28 ± 0.76^a^	2.20 ± 0.34^a^	6.80 ± 0.74^a^	2.84 ± 0.82^a^	9.64 ± 0.98^a^

^*^YS refer to yellow soil and CS calcareous soil. ^¤^Bioaccessible fraction is the supernatant solution obtained from small intestinal digestion of YS and CS cabbage and added into the Caco-2 cell monolayer. The results are expressed as mean ± SD, *n* = 3. Values for each soil within a column followed by a different letter are significantly different at *P* < 0.05, as calculated by Duncan's post hoc test. Retention or transport% is the percentages of Cd retention or transport in the total amount added (bioaccessible fraction). Cellular uptake is calculated as [(retention + transport)/total Cd amount added to cell culture (bioaccessible fraction)] × 100.

**Table 3:** Toxicity assays: lipid peroxidation (MDA), oxidant enzyme (H_2_O_2_), and antioxidant enzymes (SOD, GPx) activities in HL-7702 cells treated with different concentrations of Cd transported from human epithelial (Caco-2) cells.

Toxicity assays	Chinese cabbage	Cd concentrations in Soil (mg kg^−1^)			
		CK	2	6	9
MDA (nmol mg cell protein^−1^)	YS^*^ cabbage	19.64 ± 3.16^c^	21.41 ± 2.30^bc^	27.53 ± 5.10^ab^	30.92 ± 1.72^a^
	CS^*^ cabbage	16.40 ± 2.04^b^	17.78 ± 2.57^ab^	18.93 ± 1.62^ab^	21.03 ± 1.49^a^

H_2_O_2_ (mmol mg cell protein^−1^)	YS cabbage	134.08 ± 9.22^c^	164.56 ± 12.01^bc^	190.01 ± 20.56^ab^	223.66 ± 23.17^a^
	CS cabbage	118.94 ± 19.25^b^	130.10 ± 10.41^b^	157.50 ± 15.34^ab^	180.54 ± 18.43^a^

SOD (U mg cell protein^−1^)	YS cabbage	26 ± 4.08^a^	23.11 ± 2.06^a^	19.06 ± 4.63^ab^	14.69 ± 4.22^b^
	CS cabbage	26.88 ± 3.24^a^	27.53 ± 4.83^a^	26.15 ± 4.20^a^	21.71 ± 3.70^a^

GPx (U mg cell protein^−1^)	YS cabbage	57.42 ± 3.00^a^	53.61 ± 2.20^ab^	47.65 ± 4.32^b^	35.68 ± 4.17^c^
	CS cabbage	63.81 ± 5.21^a^	62.49 ± 2.37^a^	58.53 ± 4.16^a^	50.34 ± 1.79^b^

^*^YS refer to yellow soil and CS calcareous soil.  The results are expressed as mean ± SD,  *n* = 3. Values within a row followed by a different letters are significantly different at *P* < 0.05, as calculated by Duncan's post hoc test.
